# Paraspinal Rhabdomyolysis: A Rare but Essential Diagnosis With a Back Pain Bounceback

**DOI:** 10.7759/cureus.95905

**Published:** 2025-11-01

**Authors:** David A Wilson, Lindsay M Martin, Ashley N Kim, Tristan J Baltazor, Joshua J Oliver

**Affiliations:** 1 Department of Emergency Medicine, Madigan Army Medical Center, Tacoma, USA

**Keywords:** acute low back pain, common emergency department complaints, emergency department, emergency department bouncebacks, exercise-induced rhabdomyolysis, exertional rhabdomyolysis, low back pain (lbp), pain in emergency department, paraspinal muscles, trigger point injections

## Abstract

Paraspinal rhabdomyolysis is a rare but important diagnosis in the emergency department (ED). Diagnosis is challenging due to the difficulty in identifying cardinal signs of "pain out of proportion" on exam that differ from the common complaint of muscular back pain. Moreover, exertional rhabdomyolysis is less common than drug- or heat-induced rhabdomyolysis, and these patients are often young, male, endurance athletes. This report adds to the literature with a case of exertional paraspinal rhabdomyolysis in an overweight woman, not exposed to heat or medications predisposing to rhabdomyolysis, and whose pain was initially responsive to trigger point injections.

A 38-year-old woman with a past medical history of polycystic ovary syndrome presented to the ED with low back pain, having presented the previous day with the same symptoms after completing an intense physical workout four days prior to initial presentation. Due to presumed musculoskeletal soreness/spasm as the etiology of her symptoms, she was again treated with trigger point injections and over-the-counter analgesia with moderate improvement in her pain, consistent with her course the previous day. Further evaluation was indicated given the patient's repeat presentation, which demonstrated paraspinal rhabdomyolysis without findings concerning for compartment syndrome. The patient was treated with IV fluids and admitted for management in line with general consensus treatment. Her symptoms improved and she was discharged on hospital day 2.

Paraspinal exertional rhabdomyolysis is rare, but should be considered in patients presenting with low back pain after increased activity that is not responsive to conservative management.

## Introduction

Rhabdomyolysis is a condition resulting from the release of intracellular contents into the systemic circulation following the breakdown and necrosis of muscle tissue. It is difficult to determine its incidence due to underreporting and lack of prospective studies, but it is best characterized as an infrequent diagnosis in the emergency department (ED) [1,2]. Causes of rhabdomyolysis include direct trauma, crush injury, medication or toxic ingestion, extreme heat exposure, and, less commonly, strenuous physical activity. Most often, these symptoms are experienced in the extremities, especially the anterior compartment of the lower leg [1,3]. Electrolyte abnormalities due to intracellular content release can have secondary effects such as cardiac instability, though the most serious sequelae include acute kidney injury and compartment syndrome. Damaged myocytes release both creatine kinase (CK) and aspartate aminotransferase (AST) as part of the disease process, which can be measured to facilitate diagnosis. There is no universally accepted definition of rhabdomyolysis, but the diagnostic criterion is often cited as CK five times the upper limit of normal (>1000-1500 U/L depending on laboratory assay) [1,3,4]. Patients experiencing rhabdomyolysis can present with a wide array of symptoms such as nausea, vomiting, abdominal pain, myalgias, tenderness to palpation of muscle groups, weakness, fatigue, and low-grade fever. Due to the excretion of myoglobin by the kidneys, patients may present with dark-colored urine and urinalysis positive for hematuria without red blood cells (RBCs).

Although low back pain is a common presentation to the ED, paraspinal rhabdomyolysis as a cause has been reported in the literature in only a dozen cases over the last 20 years, with a smaller subset categorized as exertional in nature. Previous cases have been predominantly male patients with active lifestyles and with pain that was not responsive to treatment [5-7]. Since most acute and subacute low back pain resolves on its own, the American College of Physicians recommends conservative, non-pharmacological treatment followed by non-steroidal anti-inflammatory drugs (NSAIDs) with the addition of physical therapy for chronic pain [8]. Trigger point injections (TPI) have been demonstrated over multiple studies to provide relief for myofascial pain compared to NSAIDs and physical therapy alone [9].

## Case presentation

A 38-year-old woman presented to the ED in September 2023 with low back pain, having presented the previous day with the same symptoms. She had a past medical history significant for polycystic ovary syndrome and was making efforts to improve her health by committing to exercise more often in hopes of improving the chance of conception. Approximately four days prior to initial presentation, she exercised for the first time, for a duration of three hours, after which she developed severe back pain unrelieved by over-the-counter medications such as acetaminophen and ibuprofen. She presented to the ED and was evaluated for intact reflexes, appropriate lower extremity strength, absence of midline spinal tenderness, and absence of symptoms or history such as fever, malignancy, other immunocompromised state, steroid use, IV drug use, perineal anesthesia, fecal/urinary incontinence, or urinary retention that might indicate spinal epidural abscess or other infection, bony abnormality, pyelonephritis, nephrolithiasis, or symptoms consistent with spinal cord compression [3,8]. After determining that the afebrile and hemodynamically stable patient did not require antibiotics, emergent surgical intervention, or treatment to prevent significant morbidity, she was assessed to have muscle strain. Trigger points were identified in her left lower back, and after TPI of lidocaine, her pain improved, and she was discharged with lidocaine patches and continued over-the-counter conservative measures. She returned the following day with a re-presentation of her pain. She was again noted to have symptoms consistent with muscular low back pain with identifiable trigger points and no "red flags" for surgical emergency or serious infection. She received TPI for a second time using a bupivacaine and ketorolac which improved her pain from 10/10 to 5/10. However, given her presentation twice in two days, additional labs were obtained (Table 1 and Table 2).

**Table 1:** Initial serum laboratory evaluation ED: emergency department; WBC: white blood cell; CO2: carbon dioxide; BUN: blood urea nitrogen; ALT: alanine aminotransferase; AST: aspartate aminotransferase; CK: creatine kinase

Laboratory test	ED value	Reference range
WBC	12.1×10^3^/μL	4.5-13.0×10^3^/μL
Hemoglobin	13.8 g/dL	10.0-15.0 g/dL
Hematocrit	38%	34-45%
Platelets	242×10^3^/μL	140-420×10^3^/μL
Sodium	138 mmol/L	135-145 mmol/L
Potassium	4.0 mmol/L	3.5-5.1 mmol/L
Chloride	101 mmol/L	98-107 mmol/L
CO2	22 mmol/L	22-31 mmol/L
BUN	6 mg/dL	6-23 mg/dL
Creatinine	0.60 mg/dL	0.50-1.00 mg/dL
Bilirubin total	2.00 mg/dL	0.15-1.20 mg/dL
Bilirubin direct	0.4 mg/dL	0.0-0.3 mg/dL
ALT	159 U/L	0-33 U/L
AST	451 U/L	0-35 U/L
CK	33,005 U/L	26-192 U/L

**Table 2:** Initial urine laboratory evaluation UA: urinalysis; ED: emergency department; WBC: white blood cell; RBC: red blood cell

Laboratory test	ED value	Reference range
UA color	Yellow	None
UA appearance	Clear	None
UA pH	5.0	5.0-9.0
UA specific gravity	1.064	1.001-1.030
UA glucose	Negative	Negative
UA ketones	40 mg/dL	0 mg/dL
UA blood	Trace	None
UA protein	Negative	Negative
UA bilirubin	Negative	Negative
UA nitrite	Negative	Negative
UA leuk esterase	Negative	Negative
UA WBC	1/HPF	0-4/HPF
UA RBC	0/HPF	0-8/HPF
UA bacteria	Negative	Negative
UA epi squam	2/HPF	0-3/HPF

**Figure 1:**
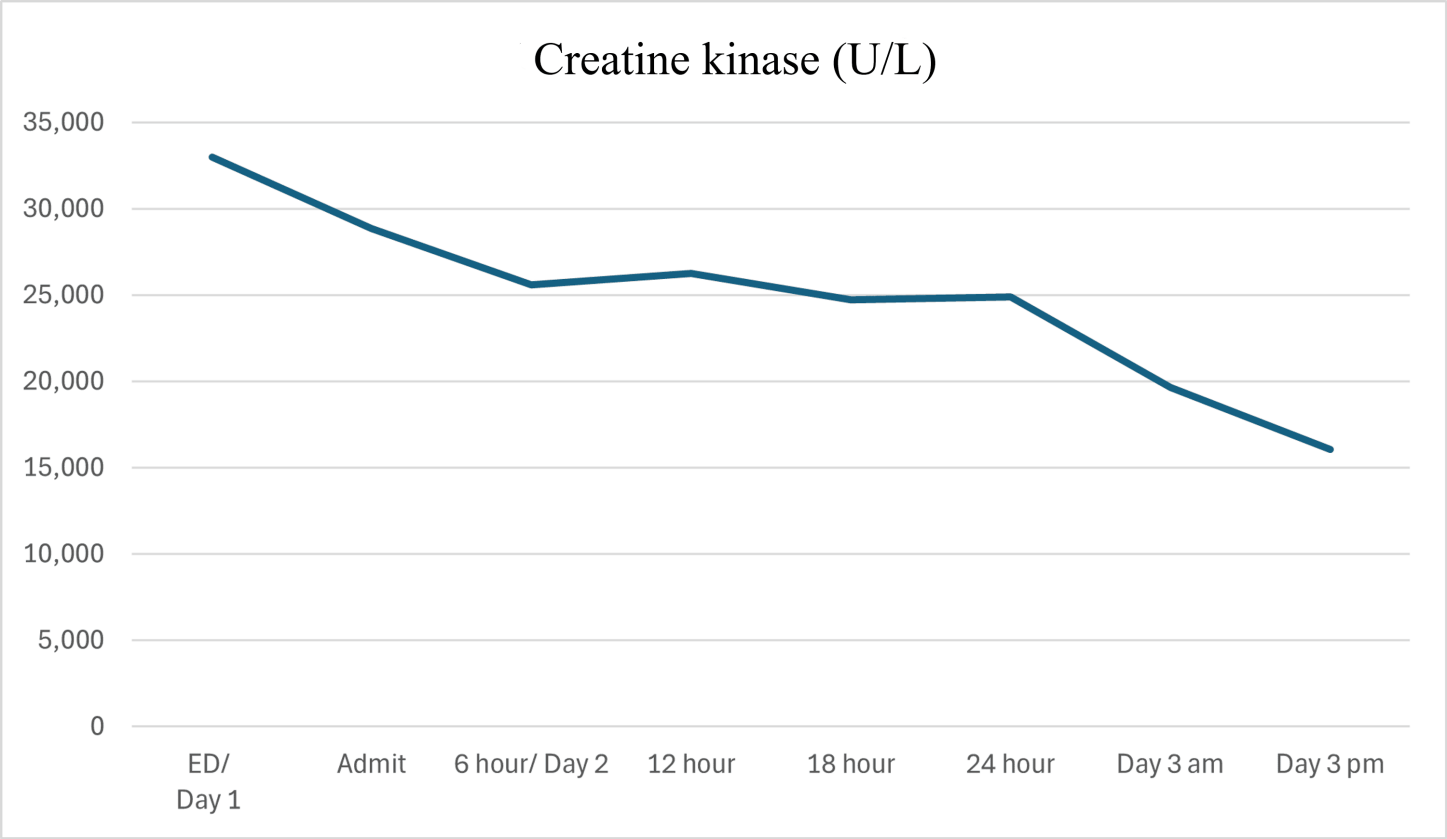
Patient's creatine kinase levels ED: emergency department

Initial lab results of elevated transaminases with AST predominance in the absence of substance use, elevated bilirubin, as well as urinalysis with trace blood without RBCs raised concern for rhabdomyolysis. The CK level was subsequently found to be 33,005 U/L. Due to the patient's body habitus, the ability to confidently palpate the paraspinal muscles to assess for firm compartments was limited. Neurosurgery was consulted for evaluation and found low concern for compartment syndrome. The patient was treated with 3L IV fluids in the ED while awaiting admission. She was admitted for continued observation, serial examinations for the development of compartment syndrome, hydration, and pain control. After two days of successful treatment, the patient demonstrated a continually down-trending CK and was discharged (Figure 1). This treatment is in line with the Eastern Association for the Surgery of Trauma guidelines and general consensus treatment [4,10].

## Discussion

This case adds to the literature by presenting a rare instance of exertional paraspinal rhabdomyolysis in a woman with no prior athletic training and no predisposing exposure to heat, medications, or trauma, whose pain was initially responsive to TPI. Existing literature mostly describes typical presentations, where rhabdomyolysis often affects young, fit men undergoing strenuous physical activity, particularly in hot environments or after exposure to certain medications [1,2]. Still, strenuous weightlifting is a recognized cause, and these presentations have grown more common with the popularity of Olympic-style weightlifting among otherwise untrained individuals [6,11,12]. Reports also highlight extremity involvement such as in the arms or thighs as the most common site of muscle breakdown in exertional rhabdomyolysis [1,2]. In contrast, paraspinal involvement remains relatively underreported. This disparity may reflect both the rarity of paraspinal rhabdomyolysis and the diagnostic challenges it presents. The nonspecific nature of back pain, combined with the difficulty of recognizing classic signs of rhabdomyolysis such as "pain out of proportion" with movement or touch in the axial skeleton, especially in patients with a larger body habitus, makes this condition easier to miss in clinical practice.

Some case reports have implicated injections (e.g., intramuscular injections, vaccines) as possible triggers for rhabdomyolysis [5]. However, in this case, the chronology of symptoms and the stable clinical picture before and after TPI suggest that injections were not the primary cause but rather a coincidental intervention during an evolving rhabdomyolysis process.

Furthermore, this case also illustrates a key gap in current diagnostic practices. The initial presentation of discrete muscular trigger points transiently improved after conservative interventions, and the absence of systemic symptoms suggested common mechanical or myofascial back pain, which may explain why rhabdomyolysis was not considered during the first ED visit. This aligns with literature indicating that rhabdomyolysis often goes unrecognized when it presents outside the classic triad of muscle pain, weakness, and dark urine, especially when patients present early in the disease process or with atypical muscle group involvement [1,2].

Importantly, this case reinforces the critical role of comprehensive discharge planning and return precautions. While the initial management provided reasonable and appropriate treatment for presumed muscular back pain, it was the patient's adherence to return instructions that enabled the timely recognition of the true etiology and allowed for definitive intervention [8]. This underscores a point echoed in the literature: that clinical vigilance and patient education are key to catching potentially serious conditions masked as benign musculoskeletal complaints.

## Conclusions

While rare, exertional rhabdomyolysis of the paraspinal muscles should be considered in patients presenting to the ED with low back pain following strenuous activity, especially if it is not responsive or only transiently responsive to conservative management. Rhabdomyolysis, and specifically paraspinal disease, is a challenging diagnosis when risk factors are present and even more so when evaluating atypical patients. Patients with refractory pain in the setting of increased activity should be considered for urinalysis, a metabolic panel, and a CK level to screen for rhabdomyolysis. Finally, comprehensive return precautions are essential to aid patients in identifying truly concerning symptoms that need re-evaluation and can help providers by prompting further opportunities to investigate rare presentations.
